# COVID-19 Pandemic: Therapeutic Strategies and Vaccines

**DOI:** 10.3390/ijms25010556

**Published:** 2023-12-31

**Authors:** Mariarosaria Boccellino

**Affiliations:** Department of Precision Medicine, University of Campania “Luigi Vanvitelli”, 80138 Naples, Italy; mariarosaria.boccellino@unicampania.it

## 1. Introduction

Severe acute respiratory syndrome Coronavirus 2 (SARS-CoV-2), a highly pathogenic and transmissible virus, has spurred an impressive accumulation of knowledge [1,2,3]. The COVID-19 pandemic has triggered profound global social and economic impacts [4,5]. Stringent lockdown measures have disrupted daily life, leading to disturbances in employment, commerce, and education [6,7]. The pandemic has exacerbated existing inequalities in access to healthcare, with vulnerable populations facing heightened challenges [8,9]. 

The emergence of a new threat has sparked a heightened focus on the advancement of rapid diagnostic methods for respiratory viral infections [10,11]. A key challenge lies in differentiating between various respiratory viruses, including SARS-CoV-2, influenza, adenovirus, and respiratory syncytial virus [12,13]. These viruses can induce similar clinical symptoms such as fever, chills, cough, shortness of breath, fatigue, sore throat, and headache [14,15].

The evolving understanding emphasizes the pivotal role of robust immunity as the primary defense against COVID-19 [16,17]. The constant emergence of SARS-CoV-2 variants adds complexity to the infection landscape, influencing both the extent of viral spread and the efficacy of immune responses, whether acquired through infection or induced by vaccination [18,19,20,21]. Additionally, host genetics and gene expression regulation have emerged as crucial factors shaping individual responses to the infection [22,23]. The genetic landscape of host individuals plays a pivotal role in determining susceptibility, severity of infection, and response to therapeutic interventions [24,25]. Gene expression regulation adds another layer of complexity, highlighting the intricate ways in which our molecular makeup influences the course of the disease [26,27].

In the realm of therapeutic exploration, a diverse array of treatments has been evaluated [28,29]. From conventional measures such as oxygen therapy and corticosteroids to antiviral agents, targeted therapies, and vaccines, each avenue presents a unique set of effects and outcomes, reflecting the heterogeneity of the patient population [30,31].

However, amid the diversity of therapeutic approaches, no singular option has emerged as a universally approved remedy for COVID-19. This underscores the multifaceted nature of the disease, with varied responses and outcomes observed in different individuals.

While traditional treatments continue to be explored, recent in vitro and in vivo studies have opened new frontiers. Novel approaches, including gene editing, cell-based therapy, and immunotherapy, hold significant promise for reshaping the landscape of COVID-19 treatment [32]. As we navigate the dynamic challenges posed by this pandemic, a comprehensive understanding of the interplay between the virus, host factors, and evolving therapeutic modalities becomes increasingly critical [33,34,35].

This Special Issue provides a comprehensive overview of the diverse studies centered around the COVID-19 pandemic. It includes nine research articles, three communications, three reviews, and one brief report, each offering unique insights into various aspects of the pandemic’s impact. The main purpose of this editorial is to provide readers with a quick and clear overview of the most significant contributions presented, inviting them to delve further into these topics to enrich their critical understanding of the scientific context and practical implications related to COVID-19.

## 2. An Overview of Published Articles

Contradictory reports exist regarding the potential impact of vaccines on the immune system, particularly in terms of inducing the production of autoantibodies. The role of natural autoantibodies (nAAbs) within this context remains insufficiently defined. A hypothesis is put forth suggesting that physiological nAAbs might exhibit a degree of adaptability when stimulated by immunological triggers. To investigate this hypothesis, Szinger et al. conducted a study using a sample from Croatia, involving both antiviral antibodies (anti-MMR and anti-SARS-CoV-2 IgG) and nAAbs (specifically, anti-citrate synthase IgG and IgM) [36]. The study included individuals with documented vaccination histories. Remarkably, the findings reveal a statistically significant association between IgM isotype nAAbs and anti-MMR IgG seropositivity. Furthermore, IgG isotype nAAbs showed elevated levels in individuals with anti-SARS-CoV-2 antibodies and in those who received a mixed vaccine regimen. These results suggest an interaction between immune activation and the dynamic expansion of nAAbs. Consequently, it is proposed that a reassessment of the influence of nAAbs on immunization effectiveness and their role in the intricate functioning of the immune system is warranted.

As vaccination campaigns continue to combat SARS-CoV-2, there remains an urgent need for effective antiviral treatments, especially for individuals with more severe disease courses. Recently, significant efforts have been made to repurpose existing drugs as potential antiviral agents. One such drug under investigation is the local anesthetic procaine, which has been studied for its antiviral properties against various viruses in the past. Clio Häring et al. in their study, present data on the inhibitory effects of two procaine prodrugs, ProcCluster^®^ and procaine hydrochloride, on SARS-CoV-2 infection in vitro [37]. Both of these procaine prodrugs demonstrate the ability to reduce SARS-CoV-2 progeny virus titers while also diminishing interferon and cytokine responses in a manner that is proportional to the viral load. Notably, when procaine is added during the early stages of the SARS-CoV-2 replication cycle in cell culture, it initially restricts the production of subgenomic RNA transcripts and subsequently impacts the replication of the viral genomic RNA. Interestingly, procaine continues to exert a significant effect on the release of SARS-CoV-2 progeny viruses, even when added late in the replication cycle, a point at which viral RNA and protein production are largely completed. 

The COVID-19 pandemic continues to impact the world with the emergence of new contagious variants. While vaccination is the most effective means of prevention, limited access to vaccines in some countries remains a concern due to manufacturing and transportation constraints. To address this issue, researchers have developed an affordable and easy-to-use vaccination approach using genetically modified microorganisms, particularly probiotics. In the study by Chau et al., Lactobacillus casei was chosen as an oral vaccine candidate because of its natural immunoadjuvant properties and ability to survive the stomach’s acidic environment [38]. This modified probiotic was engineered to express SARS-CoV-2 Omicron variant B.1.1.529 antigens with B-cell and T-cell epitopes. The resulting vaccine, named OMGVac, successfully triggered a strong IgG immune response against the Omicron variant in Golden Syrian hamsters. Importantly, no adverse effects were observed, and the vaccine’s safety was confirmed through physiological and histopathological examinations of various organs. This study also demonstrated the potential of using recombinant probiotics as live delivery vectors to initiate systemic immunity, offering promising prospects for developing next-generation vaccines against emerging infectious diseases. 

The COVID-19 pandemic, caused by the highly infectious SARS-CoV-2 virus, necessitates precise measurement of neutralizing antibodies to assess the effectiveness of mitigation efforts like vaccination and monoclonal therapeutics. To achieve this, the development of rapid, safe, and user-friendly neutralization assays is crucial for swift diagnosis and treatment. In this regard, Izac et al. created a neutralization assay based on the vesicular stomatitis virus (VSV) with two assessment methods, imaging and flow cytometry, to quantify varying levels of neutralization in patient serum samples [39]. They tested two different spike-pseudoviruses and optimized the assay by conducting time-course experiments at different infection levels. The results from this assay align with previously established serology and surrogate neutralization assays. The two pseudovirus assessment methods yielded similar 50% neutralization titer values. The use of in situ readouts for live-cell imaging and high-throughput analysis with flow cytometry offers unique capabilities for the rapid assessment of neutralization, a critical factor in addressing future pandemics.

Human herpesviruses (HHVs) are known for their ability to establish latency and cause neurological disorders. HHV-6, a herpesvirus, has been associated with such disorders. Some studies have reported finding HHV-6 in COVID-19 patients with neurological symptoms. However, diagnosing these neurological disorders caused by the viruses can be invasive and challenging. The study by Carneiro et al. aimed to establish a connection between microRNAs (miRNA) and neurological symptoms in patients co-infected with COVID-19 and HHV-6, as well as to explore the potential of miRNAs as biomarkers [40]. The study analyzed serum samples from three groups of COVID-19 patients. Real-time polymerase chain reaction (qPCR) was used to examine miRNA levels associated with neuroinflammation in patients with neurological issues and HHV-6 detection. A comparison between the group of patients without HHV DNA detection and neurological problems and the group with HHV-6 DNA detection and neurological issues revealed significant differences in the expression of specific miRNAs (mir-21, mir-146a, miR-155, and miR-let-7b). These findings emphasize the role of miRNAs in neurological disorders and suggest their potential as biomarkers for conditions triggered by HHV-6. Additionally, understanding miRNA expression may contribute to the development of therapeutic strategies.

The study by Yoshizue et al. examined the potential of using *Escherichia coli*-expressed proteins as antigens for subunit vaccines [41]. They focused on the *E. coli*-expressed SARS-CoV-2 receptor-binding domain (RBD) of the spike protein as a model. The goal was to determine if it could generate neutralizing antibodies comparable to those produced by the S1 subunit of the spike protein expressed in mammalian cells. They conducted immunization experiments on 5-week-old female mice using various injection schemes: two injections of RBD (30 µg) with an 8-week interval (RBD/RBD); two injections of S1 subunit (5 µg) with no interval (S1/S1); one injection of RBD followed by one injection of S1 subunit (RBD/S1); one injection of S1 subunit followed by one injection of RBD (S1/RBD). Ten weeks after the initial injection (two weeks after the second injection), all injection combinations resulted in a robust immune response, with IgG titers exceeding 105 (S1/RBD < S1/S1 < RBD/S1 < RBD/RBD). Furthermore, the neutralization effectiveness of the sera ranked as follows: S1/RBD and RBD/S1 (80%) > S1/S1 (56%) > RBD/RBD (42%). These findings suggest that two injections of *E. coli*-expressed RBD or mammalian-cell-produced spike S1 subunit alone can offer some protection against SARS-CoV-2, but a mixed injection scheme provides significantly higher protection. 

The challenge posed by rapidly evolving SARS-CoV-2 variants and their impact on COVID-19 vaccines is being addressed. Traditional virus neutralization tests offer complex data on the neutralization of different variants, making it difficult to assess the breadth of the antibody response. To address this issue, an antigenic cartography approach was used in the study by Astakhova et al. The immune responses of individuals who received the Sputnik V booster vaccine after their initial Sputnik V vaccination were compared with those who received Comirnaty (Pfizer-BioNTech) boosters [42]. Traditional analysis indicated robust humoral responses against various SARS-CoV-2 variants, including the wild-type, Alpha, Beta, Delta, Omicron BA.1, and BA.4/BA.5 variants, after both homologous and heterologous booster vaccinations. However, a more detailed analysis using antigenic cartography revealed that the Omicron variants remained antigenically distant from the wild-type strain. This suggests that the levels of cross-neutralizing antibodies generated may be insufficient. These findings have important implications for the development of new vaccine regimens, highlighting the specific challenges posed by Omicron variants. 

The study by Trofin et al. aimed to examine the serum concentrations of interleukin-6 (IL-6), C-reactive protein (CRP), D-dimer, lactate dehydrogenase (LDH), ferritin, and procalcitonin in COVID-19 patients with varying disease severity [43]. A prospective cohort study was conducted, involving 137 consecutive COVID-19 patients, who were categorized into four groups based on disease severity: 30 patients with mild symptoms, 49 with moderate symptoms, 28 with severe symptoms, and 30 with critical symptoms. The study examined how the tested parameters correlated with the severity of COVID-19. The results showed significant differences in COVID-19 severity based on vaccination status, LDH concentrations depending on the virus variant, and IL-6, CRP, and ferritin concentrations in relation to gender and vaccination status. Receiver Operating Characteristic (ROC) analysis identified D-dimer as the most reliable predictor of severe COVID-19 forms, and LDH as a predictor of the virus variant. The findings confirmed the connection between inflammation markers and the clinical severity of COVID-19, with all biomarkers increasing in cases of severe and critical COVID-19. IL-6, CRP, ferritin, LDH, and D-dimer levels were elevated in all forms of COVID-19. Notably, these inflammatory markers were lower in patients infected with the Omicron variant. The study also highlighted that unvaccinated patients were more likely to develop severe forms of the disease, leading to a higher hospitalization rate. D-dimer emerged as a useful predictor for severe COVID-19, while LDH could predict the virus variant.

Ruggieri’s article focuses on characterizing the immune responses, including antibodies and T-cells, in patients with multiple sclerosis (PwMS) who are receiving various disease-modifying treatments (DMTs) after receiving COVID-19 vaccines [44]. A total of 134 PwMS and 99 healthcare workers (HCWs) who had completed the two-dose COVID-19 mRNA vaccine schedule within the last 2–4 weeks (T0) were prospectively enrolled. They were followed up at 24 weeks after the first dose (T1) and 4–6 weeks after receiving the booster dose (T2). Results indicated that PwMS experienced a significant decrease in the seroconversion rate and anti-receptor-binding domain (RBD) immunoglobulin (IgG) titers from T0 to T1. However, there was a significant increase in these parameters from T1 to T2. The booster dose in PwMS led to a substantial improvement in the serologic response, even surpassing that of HCWs. It resulted in a significant five-fold increase in anti-RBD-IgG titers compared to T0. Similarly, the T-cell response showed a significant 1.5- and 3.8-fold increase in PwMS at T2 compared to T0 and T1, respectively, without significant changes in the number of responders. Notably, regardless of the time elapsed since vaccination, a majority of ocrelizumab-treated patients (77.3%) exhibited a T-cell-specific response, while fingolimod-treated patients (93.3%) predominantly showed a humoral-specific response. The booster dose significantly reinforced both humoral and cell-mediated immune responses. It also highlighted specific immune weaknesses induced by certain DMTs, emphasizing the importance of tailoring strategies for immunocompromised patients. These strategies should include primary prophylaxis, early SARS-CoV-2 detection, and timely management of COVID-19 antiviral treatments. 

In response to the ongoing global outbreaks of infectious diseases, there’s a growing need for fast and dependable virus identification methods. Various techniques have been deployed to screen and diagnose patients. Polymerase chain reaction with reverse transcription is highly reliable and sensitive but not particularly rapid. On the other hand, antibody-based strips are fast but may not always be reliable or sensitive. To address these challenges, a range of alternative tools are under development to cater to diverse customer needs. One such innovation is surface-enhanced Raman spectroscopy (SERS), which offers the potential for single-molecule detection within a matter of minutes. In their study, Kukushkin et al. developed a multiplex lithographic SERS aptasensor with the aim of simultaneously detecting multiple respiratory viruses in a single test, completed in just 17 minutes [45]. This aptasensor incorporates four aptamers, each labeled differently, and they are anchored onto the metal surface of four distinct SERS zones. When viruses are captured by these aptamers, they influence the SERS signals of the labels, resulting in detectable changes in the analytical signals. Notably, this sensor can effectively identify a mixture of respiratory viruses, including SARS-CoV-2, influenza A virus, respiratory syncytial virus, and adenovirus, all within a single experiment using a one-step recognition process. This innovative approach streamlines and expedites the detection of multiple viruses, which is crucial for timely and accurate diagnoses, especially during outbreaks. 

In a study by Lasagna et al., the aim was to understand the role and longevity of the immune response generated by the BNT162b2 mRNA vaccine against SARS-CoV-2 in cancer patients one year after receiving the third vaccine dose [46]. Researchers prospectively assessed the long-term immune response of 55 patients undergoing active cancer treatment. They measured neutralizing antibody (NT Ab) levels against Omicron variants and total anti-trimeric S IgG levels one year after the third vaccine dose. Additionally, they evaluated the T-cell response against the spike protein. The findings revealed that 67.3% of the patients had positive total anti-trimeric S IgG levels one year after the third dose. Regarding T-cell responses, the frequency of responder patients remained stable between six and twelve months after the third dose. Importantly, less than 20% of cancer patients had undetectable NT Ab titers against Omicron variants of concern (VOCs). Notably, the type of underlying cancer therapy did not appear to significantly impact the magnitude or frequency of the immune response. Overall, the study by Lasagna and colleagues underscores the persistence of both humoral and cellular immune responses to the BNT162b2 vaccine in a cohort of cancer patients one year after receiving the third dose, irrespective of their specific cancer treatment. This information is important for understanding the vaccine’s effectiveness and durability in a population with unique healthcare needs. 

In the study conducted by Lucca et al., the focus was on assessing the immunogenicity and safety of the BNT162b2 vaccine in a cohort of 260 individuals with cystic fibrosis (pwCF), which included 18 lung transplant recipients (LTR) [47]. Cystic fibrosis is known for its progressive decline in lung function, and viral infections can exacerbate this condition. As CF is considered a comorbidity for COVID-19, there has been a proposal to prioritize SARS-CoV-2 vaccination for pwCF. Additionally, lung transplant recipients have demonstrated poor outcomes after SARS-CoV-2 infections, and their immunization response to the mRNA-based BNT162b2 vaccine, especially when undergoing immunosuppressive treatments like mycophenolate mofetil (MMF) therapy, has raised concerns. The study aimed to determine the immunogenicity of the BNT162b2 vaccine in pwCF, focusing on serum levels of neutralizing anti-SARS-CoV-2 IgG and IgA antibodies following the administration of two vaccine doses. The results indicated that pwCF exhibited a vaccine-induced IgG and IgA antiviral response comparable to that observed in the general population. However, the immunogenicity of the BNT162b2 vaccine was significantly reduced in the subset of lung transplant recipients, particularly in those receiving MMF therapy. Despite these variations in immune response, the BNT162b2 vaccine’s safety profile in pwCF was generally consistent with that of the general population, with minor adverse events reported, mostly after the second vaccine dose. The study’s findings support the use of the BNT162b2 vaccine in pwCF and emphasize the importance of ongoing assessments of anti-SARS-CoV-2 IgG and IgA neutralizing antibody responses to COVID-19 vaccination in this patient population. 

In the review by Guo et al., the profound impact of the COVID-19 pandemic is discussed, extending beyond respiratory issues and affecting various organs and functions [48]. In severe cases, the disease can progress to acute respiratory distress syndrome (ARDS) and multi-organ failure, often driven by an excessive immune response known as a cytokine storm. In this context, mesenchymal stem cells (MSCs) have garnered attention due to their potential to alleviate inflammation, modulate immune responses, and facilitate tissue regeneration. Accumulating evidence supports the effectiveness and safety of MSCs in treating severe COVID-19 and ARDS. However, critical aspects, including optimal routes of MSC administration, appropriate dosage, treatment intervals, management of extrapulmonary complications, and potential applications in pediatric cases, require further investigation. This review, authored by Guo et al., holds promise for enriching our understanding and refining the application of MSCs in addressing the multifaceted challenges posed by COVID-19. 

SARS-CoV-2 vaccination has offered a pathway out of the pandemic and its global health, social, and economic impacts. However, in addition to vaccine efficacy, safety is a crucial concern. While mRNA-based vaccines are generally considered safe, there have been a growing number of reported side effects as more people worldwide receive these vaccines. Myopericarditis is a notable cardiovascular complication associated with these vaccines, but other side effects should not be underestimated. The review by Cocco et al. presents a series of cases involving patients who experienced cardiac arrhythmias following mRNA-based COVID-19 vaccination, drawing from both clinical practice and existing literature [49]. Analysis of official vigilance databases reveals that heart rhythm disorders after COVID vaccination are not uncommon and warrant greater clinical and scientific attention. Despite the overall favorable risk-benefit ratio of vaccination, cardiac arrhythmias are a significant concern, with emerging indications in the literature suggesting a risk of post-vaccination malignant arrhythmias in predisposed individuals. Given these findings, the review delves into potential molecular pathways by which COVID-19 vaccines could impact cardiac electrophysiology and lead to heart rhythm disorders. This exploration highlights the importance of closely monitoring and addressing potential cardiovascular side effects in the ongoing vaccination efforts. 

The review by Nicolaidou et al. addresses the significant impact of the SARS-CoV-2 virus and the crucial development of effective vaccines [50]. By 12 January 2022, nine vaccines had received Emergency Use Listing from the World Health Organization, with four of them approved or authorized by the Centers for Disease Control and Prevention (CDC) in the United States. One notable gap in the initial clinical trials of COVID-19 vaccines was the exclusion of pregnant and lactating individuals, resulting in a lack of data on how the vaccines affect breast milk. Additionally, none of the authorized vaccines have been approved for use in infants under six months. Infants in their first months of life rely on antibodies from their mothers’ breast milk as a key protective mechanism since they cannot produce their own antibodies. Several studies have indicated the presence of SARS-CoV-2 antibodies in the breast milk of vaccinated or naturally infected women. However, whether these antibodies provide protection remains uncertain. Notably, research on the BNT162b2 mRNA vaccine from Pfizer-BioNTech and the mRNA-1273 vaccine from Moderna suggests that these vaccines do not release significant amounts of mRNA into breast milk, posing no risk to breastfed infants. The primary objective of this systematic review is to consolidate the current knowledge regarding the presence of immunoglobulins in human milk elicited by SARS-CoV-2 vaccines and assess their potential for virus neutralization. Additionally, the review aims to quantify the side effects experienced by lactating mothers who have been vaccinated and explore potential adverse effects on their infants. This study is pivotal as it informs decision-making by examining the antibody secretion in breast milk, particularly concerning infants’ vulnerability to COVID-19, even though the virus is generally less severe in younger individuals. 

In the brief report by Algarate et al. in the context of the RIPOVAC study, the objective was to assess the immune response in healthcare workers who received a booster dose (third dose) in terms of the strength and duration of induced antibodies [51]. During the second phase of the RIPOVAC study, which took place between December 2021 and January 2022, 389 voluntary, immunocompetent healthcare workers who were not pregnant and had previously received two vaccine doses were administered a booster dose of a SARS-CoV-2 vaccine. The study divided participants into two groups: those with and without a prior SARS-CoV-2 infection. The quantification of anti-S1 IgG (in AU/mL) was performed using CMIA (Abbott). All healthcare workers were found to be anti-S IgG positive eight months after receiving the booster dose, with an average antibody level of 17,040 AU/mL. Among the 53 participants who had not previously been infected, antibody levels increased by an average of 10,762 AU/mL, which is significantly higher than the levels generated after the second dose (1506 AU/mL). This boost in antibody levels following the booster dose is substantial and remains elevated at eight months post-boost, surpassing the levels achieved after the second dose. This suggests a potentially prolonged immunity of over one year. The study underscores the efficacy of booster doses of anti-SARS-CoV-2 vaccines, providing valuable insights into their effectiveness. 

## 3. Conclusions

This collection of articles on COVID-19 covers diverse critical topics related to the pandemic. Conflicting reports have arisen regarding the impact of COVID-19 vaccines on the immune system, especially their potential to induce the production of autoantibodies. Researchers investigated the repurposing of existing drugs, such as procaine, as antiviral agents, demonstrating in vitro inhibition of SARS-CoV-2 infection and opening a new avenue for treatment. In combating SARS-CoV-2 variants, researchers utilized diverse strategies, including *E. coli*-expressed proteins for subunit vaccines, to assess cross-neutralizing antibodies from different booster vaccines. MSCs showed promise in treating COVID-19 complications, such as inflammation and multi-organ failure, warranting further investigation into administration routes and dosages to optimize treatment approaches. Of key importance in the fight against COVID-19 are reliable and efficient virus identification methods. One innovative approach is surface-enhanced Raman spectroscopy (SERS), which offers the potential for rapid detection of multiple respiratory viruses.

In conclusion, these studies contribute valuable insights into vaccine responses, treatment approaches, safety considerations, and virus detection methods, enhancing our understanding and strategies in the ongoing battle against COVID-19.

## References

[1] Vitiello A., La Porta R., Trama U., Ferrara F., Zovi A., Auti A.M., Di Domenico M., Boccellino M. (2022). Pandemic COVID-19, an update of current status and new therapeutic strategies. Naunyn-Schmiedeberg’s Arch. Pharmacol..

[2] Han Y., Xu P., Wang Y., Zhao W., Zhang J., Zhang S., Wang J., Jin Q., Wu Z. (2023). Panoramic analysis of coronaviruses carried by representative bat species in Southern China to better understand the coronavirus sphere. Nat. Commun..

[3] Khan S., Siddique R., Shereen M.A., Ali A., Liu J., Bai Q., Bashir N., Xue M. (2020). Emergence of a Novel Coronavirus, Severe Acute Respiratory Syndrome Coronavirus 2: Biology and Therapeutic Options. J. Clin. Microbiol..

[4] Daly M., Robinson E. (2022). Depression and anxiety during COVID-19. Lancet.

[5] Wan J., Liu L., Chen Y., Zhang T., Huang J. (2023). Psychological resilience matters in the relationship between the decline in economic status and adults’ depression half a year after the outbreak of the COVID-19 pandemic. Front. Psychiatry.

[6] Muñoz-Martínez A.M., Naismith I. (2023). Social connectedness, emotional regulation, and health behaviors as correlates of distress during lockdown for COVID-19: A diary study. Appl. Psychol. Health Well Being..

[7] Canet-Juric L., Andrés M.L., Del Valle M., López-Morales H., Poó F., Galli J.I., Yerro M., Urquijo S. (2020). A longitudinal study on the emotional impact cause by the COVID-19 pandemic quarantine on general population. Front. Psychol..

[8] Odufalu F.D., Sewell J.L., Rudrapatna V., Somsouk M., Mahadevan U. (2023). Assessing the Impact of COVID-19 on IBD Outcomes Among Vulnerable Patient Populations in a Large Metropolitan Center. Inflamm. Bowel Dis..

[9] Wakeel F., Jia H., He L., Shehadeh K.S., Nappr L.E. (2023). Development and Application of a Comprehensive Measure of Access to Health Services to Examine COVID-19 Health Disparities. Healthcare.

[10] Di Domenico M., De Rosa A., Boccellino M. (2021). Detection of SARS-COV-2 Proteins Using an ELISA Test. Diagnostics.

[11] Di Domenico M., De Rosa A., Di Gaudio F., Internicola P., Bettini C., Salzano N., Castrianni D., Marotta A., Boccellino M. (2021). Diagnostic Accuracy of a New Antigen Test for SARS-CoV-2 Detection. Int. J. Environ. Res. Public Health.

[12] Groves H.E., Piché-Renaud P.-P., Peci A., Farrar D.S., Buckrell S., Bancej C., Sevenhuysen C., Campigotto A., Gubbay J.B., Morris S.K. (2021). The impact of the COVID-19 pandemic on influenza, respiratory syncytial virus, and other seasonal respiratory virus circulation in Canada: A population-based study. Lancet Reg. Health Am..

[13] Achangwa C., Park H., Ryu S., Lee M.-S. (2022). Collateral Impact of Public Health and Social Measures on Respiratory Virus Activity during the COVID-19 Pandemic 2020–2021. Viruses.

[14] Aslan A., Aslan C., Zolbanin N.M., Jafari R. (2021). Acute respiratory distress syndrome in COVID-19: Possible mechanisms and therapeutic management. Pneumonia.

[15] Han H., Saed Y.A., Song W., Wang M., Li Y. (2022). Prevalence of non-SARS-CoV-2 respiratory pathogens and co-infection with SARS-CoV-2 in the early stage of COVID-19 epidemic. Pathogens.

[16] Diamond M.S., Kanneganti T.D. (2022). Innate immunity: The first line of defense against SARS-CoV-2. Nat. Immunol..

[17] Vabret N., Britton G.J., Gruber C., Hegde S., Kim J., Kuksin M., Levantovsky R., Malle L., Moreira A., Park M.D. (2020). Immunology of COVID-19: Current State of the Science. Immunity.

[18] Chi W.Y., Li Y.D., Huang H.C., Chan T.E.H., Chow S.Y., Su J.H., Ferrall L., Hung C.F., Wu T.C. (2022). COVID-19 vaccine update: Vaccine effectiveness, SARS-CoV-2 variants, boosters, adverse effects, and immune correlates of protection. J. Biomed. Sci..

[19] Khandia R., Singhal S., Alqahtani T., Kamal M.A., El-Shall N.A., Nainu F., Desingu P.A., Dhama K. (2022). Emergence of SARS-CoV-2 Omicron (B.1.1.529) variant, salient features, high global health concerns and strategies to counter it amid ongoing COVID-19 pandemic. Environ. Res..

[20] Vitiello A., Ferrara F., Auti A.M., Di Domenico M., Boccellino M. (2022). Advances in the Omicron variant development. J. Intern. Med..

[21] Ferrara F., Mancaniello C., Varriale A., Sorrentino S., Zovi A., Nava E., Trama U., Boccellino M., Vitiello A. (2022). COVID-19 mRNA Vaccines: A Retrospective Observational Pharmacovigilance Study. Clin. Drug Investig..

[22] Karaderi T., Bareke H., Kunter I., Seytanoglu A., Cagnan I., Balci D., Barin B., Hocaoglu M.B., Rahmioglu N., Asilmaz E. (2020). Host Genetics at the Intersection of Autoimmunity and COVID-19: A Potential Key for Heterogeneous COVID-19 Severity. Front. Immunol..

[23] Niemi M.E.K., Daly M.J., Ganna A. (2022). The human genetic epidemiology of COVID-19. Nat. Rev. Genet..

[24] Papageorgiou L., Papakonstantinou E., Diakou I., Pierouli K., Dragoumani K., Bacopoulou F., Chrousos G.P., Eliopoulos E., Vlachakis D. (2023). Semantic and Population Analysis of the Genetic Targets Related to COVID-19 and Its Association with Genes and Diseases. Adv. Exp. Med. Biol..

[25] Udomsinprasert W., Nontawong N., Saengsiwaritt W., Panthan B., Jiaranai P., Thongchompoo N., Santon S., Runcharoen C., Sensorn I., Jittikoon J. (2023). Host genetic polymorphisms involved in long-term symptoms of COVID-19. Emerg Microbes Infect..

[26] Nakanishi T., Willett J., Farjoun Y., Allen R.J., Guillen-Guio B., Adra D., Zhou S., Richards J.B. (2023). Alternative splicing in lung influences COVID-19 severity and respiratory diseases. Nat. Commun..

[27] Swain J., Merida P., Rubio K., Bracquemond D., Neyret A., Aguilar-Ordoñez I., Günther S., Barreto G., Muriaux D. (2023). F-actin nanostructures rearrangements and regulation are essential for SARS-CoV-2 particle production in host pulmonary cells. iScience.

[28] Yang Y.F., Singh S. (2023). Pharmacogenomic Landscape of Ivermectin and Selective Antioxidants: Exploring Gene Interplay in the Context of Long COVID. Int. J. Mol. Sci..

[29] Leinen Z.J., Mohan R., Premadasa L.S., Acharya A., Mohan M., Byrareddy S.N. (2023). Therapeutic Potential of Cannabis: A Comprehensive Review of Current and Future Applications. Biomedicines.

[30] Fukuda Y., Mochizuki K., Ijichi M., Homma T., Tanaka A., Sagara H. (2023). Efficacy of Additional Corticosteroids After Dexamethasone Treatment for Moderate to Severe COVID-19: An Observational Study. Cureus.

[31] Pan J.Q., Tian Z.M., Xue L.B. (2023). Hyperbaric Oxygen Treatment for Long COVID: From Molecular Mechanism to Clinical Practice. Curr. Med. Sci..

[32] Shirzad M., Nourigorji M., Sajedi A., Ranjbar M., Rasti F., Sourani Z., Moradi M., Mostafa M.S., Memar M.Y. (2022). Targeted therapy in Coronavirus disease 2019 (COVID-19): Implication from cell and gene therapy to immunotherapy and vaccine. Int. Immunopharmacol.

[33] Urano E., Itoh Y., Suzuki T., Sasaki T., Kishikawa J.I., Akamatsu K., Higuchi Y., Sakai Y., Okamura T., Mitoma S. (2023). An inhaled ACE2 decoy confers protection against SARS-CoV-2 infection in preclinical models. Sci. Transl. Med..

[34] Gaynor K.U., Vaysburd M., Harman M.A.J., Albecka A., Jeffrey P., Beswick P., Papa G., Chen L., Mallery D., McGuinness B. (2023). Multivalent bicyclic peptides are an effective antiviral modality that can potently inhibit SARS-CoV-2. Nat. Commun..

[35] Rauf A., Abu-Izneid T., Khalil A.A., Hafeez N., Olatunde A., Rahman M., Semwal P., Al-Awthan Y.S., Bahattab O.S., Khan I.N. (2022). Nanoparticles in clinical trials of COVID-19: An update. Int. J. Surg..

[36] Szinger D., Berki T., Németh P., Erdo-Bonyar S., Simon D., Drenjančević I., Samardzic S., Zelić M., Sikora M., Požgain A. (2023). Following Natural Autoantibodies: Further Immunoserological Evidence Regarding Their Silent Plasticity and Engagement in Immune Activation. Int. J. Mol. Sci..

[37] Häring C., Jungwirth J., Schroeder J., Löffler B., Engert B., Ehrhardt C. (2023). The Local Anaesthetic Procaine Prodrugs ProcCluster^®^ and Procaine Hydrochloride Impair SARS-CoV-2 Replication and Egress In Vitro. Int. J. Mol. Sci..

[38] Chau E.C.T., Kwong T.C., Pang C.K., Chan L.T., Chan A.M.L., Yao X., Tam J.S.L., Chan S.W., Leung G.P.H., Tai W.C.S. (2023). A Novel Probiotic-Based Oral Vaccine against SARS-CoV-2 Omicron Variant B.1.1.529. Int. J. Mol. Sci..

[39] Izac J.R., Kwee E.J., Tian L., Elsheikh E., Gaigalas A.K., Elliott J.T., Wang L. (2023). Development of a Cell-Based SARS-CoV-2 Pseudovirus Neutralization Assay Using Imaging and Flow Cytometry Analysis. Int. J. Mol. Sci..

[40] Carneiro V.C.d.S., Moreira O.d.C., Coelho W.L.d.C.N.P., Rio B.C., Sarmento D.J.d.S., Salvio A.L., Alves-Leon S.V., de Paula V.S., Leon L.A.A. (2023). miRNAs in Neurological Manifestation in Patients Co-Infected with SARS-CoV-2 and Herpesvírus 6 (HHV-6). Int. J. Mol. Sci..

[41] Yoshizue T., Brindha S., Wongnak R., Takemae H., Oba M., Mizutani T., Kuroda Y. (2023). Antisera Produced Using an E. coli-Expressed SARS-CoV-2 RBD and Complemented with a Minimal Dose of Mammalian-Cell-Expressed S1 Subunit of the Spike Protein Exhibits Improved Neutralization. Int. J. Mol. Sci..

[42] Astakhova E.A., Morozov A.A., Byazrova M.G., Sukhova M.M., Mikhailov A.A., Minnegalieva A.R., Gorchakov A.A., Filatov A.V. (2023). Antigenic Cartography Indicates That the Omicron BA.1 and BA.4/BA.5 Variants Remain Antigenically Distant to Ancestral SARS-CoV-2 after Sputnik V Vaccination Followed by Homologous (Sputnik V) or Heterologous (Comirnaty) Revaccination. Int. J. Mol. Sci..

[43] Trofin F., Nastase E.V., Roșu M.F., Bădescu A.C., Buzilă E.R., Miftode E.G., Manciuc D.C., Dorneanu O.S. (2023). Inflammatory Response in COVID-19 Depending on the Severity of the Disease and the Vaccination Status. Int. J. Mol. Sci..

[44] Ruggieri S., Aiello A., Tortorella C., Navarra A., Vanini V., Meschi S., Lapa D., Haggiag S., Prosperini L., Cuzzi G. (2023). Dynamic Evolution of Humoral and T-Cell Specific Immune Response to COVID-19 mRNA Vaccine in Patients with Multiple Sclerosis Followed until the Booster Dose. Int. J. Mol. Sci..

[45] Kukushkin V., Ambartsumyan O., Subekin A., Astrakhantseva A., Gushchin V., Nikonova A., Dorofeeva A., Zverev V., Keshek A., Meshcheryakova N. (2023). Multiplex Lithographic SERS Aptasensor for Detection of Several Respiratory Viruses in One Pot. Int. J. Mol. Sci..

[46] Lasagna A., Cassaniti I., Arena F., Bergami F., Percivalle E., Comolli G., Sarasini A., Ferrari A., Cicognini D., Schiavo R. (2023). Persistence of Immune Response Elicited by Three Doses of mRNA Vaccine against SARS-CoV-2 in a Cohort of Patients with Solid Tumors: A One-Year Follow-Up. Int. J. Mol. Sci..

[47] Lucca F., Bezzerri V., Danese E., Olioso D., Peserico D., Boni C., Cucchetto G., Montagnana M., Tridello G., Meneghelli I. (2023). Immunogenicity and Safety of the BNT162b2 COVID-19 Vaccine in Patients with Cystic Fibrosis with or without Lung Transplantation. Int. J. Mol. Sci..

[48] Guo B.-C., Wu K.-H., Chen C.-Y., Lin W.-Y., Chang Y.-J., Lee T.-A., Lin M.-J., Wu H.-P. (2023). Mesenchymal Stem Cells in the Treatment of COVID-19. Int. J. Mol. Sci..

[49] Cocco N., Leibundgut G., Pelliccia F., Cammalleri V., Nusca A., Mangiacapra F., Cocco G., Fanale V., Ussia G.P., Grigioni F. (2023). Arrhythmias after COVID-19 Vaccination: Have We Left All Stones Unturned?. Int. J. Mol. Sci..

[50] Nicolaidou V., Georgiou R., Christofidou M., Felekkis K., Pieri M., Papaneophytou C. (2023). Detection of SARS-CoV-2–Specific Antibodies in Human Breast Milk and Their Neutralizing Capacity after COVID-19 Vaccination: A Systematic Review. Int. J. Mol. Sci..

[51] Algarate S., Serrano L., Bueno J., Herrero-Cortina B., Alvarado E., González-Barriga M.T., Ducons M., Montero-Marco J., Arnal S., Acha B. (2023). Persistence of Anti-S1 IgG against SARS-CoV-2 Eight Months after the Booster Dose of Vaccine in Naive and Previously Infected Healthcare Workers. Int. J. Mol. Sci..

